# Evaluation of longitudinal passive immunity transfer against lumpy skin disease virus in calves by different serological methods

**DOI:** 10.1007/s11259-024-10421-0

**Published:** 2024-05-23

**Authors:** Milena Samojlović, Tamaš Petrović, Vladimir Polaček, Diana Lupulović, Gospava Lazić, Dragan Rogan, Sava Lazić

**Affiliations:** 1 Scientific Veterinary Institute “Novi Sad”, Novi Sad, Serbia; 2 Vetpro doo, Laboratory for Veterinary Clinical Diagnostics, Belgrade, Serbia; 3 Department of Veterinary Medicine, Faculty of Agriculture, University of Novi Sad, Novi Sad, Serbia

**Keywords:** Lumpy skin disease, Passive immunity, Vaccination, Antibodies, VNT, ELISA

## Abstract

To implement effective lumpy skin disease (LSD) control measures, such as timely vaccination, particularly in calves and serological monitoring, it is necessary to evaluate immune response after vaccination, both in adult cattle and in their calves. The aim of this study was to evaluate passive immunity transfer and duration of maternal antibodies against lumpy skin disease virus (LSDV) in calves born to vaccinated cows by two different serological methods. The longitudinal study was carried out on two farms in Serbia where no cases were reported during LSD outbreak in 2016. Fifteen cows on each farm were vaccinated and revaccinated with attenuated vaccine - Neethling strain. A total of 30 cows and 30 calves on both farms were included in the study. Serum samples from cows were collected on calving day and serum samples from their respective calves on days 10, 20, 30, 45, 60, 75, 90, 105 and 120 after birth. Colostrum samples were collected only from 15 cows on one farm. In order to determine the presence of antibodies against LSDV a total of 30 cow sera samples, 15 colostrum samples and 270 calf sera samples were examined by commercial enzyme-linked immunosorbent assay (ELISA) and modified virus neutralization test (VNT). Overall, the performance of both serological tests was very satisfactory. The results of this longitudinal study showed that persistence of passive immunity in calves is less than 4 months, and that most calves are not protected against LSDV at that age. Since the vaccination is the most important control measure against LSDV, the recommended age of six months for vaccination of calves born to vaccinated cows should be reassessed to achieve the most optimal protection against LSD.

## Introduction

Lumpy skin disease is a highly contagious viral disease of cattle. Due to its economic importance and ability to spread rapidly the disease is listed under World Organization for Animal Health (WOAH) notifiable diseases of cattle (EFSA 2015; WOAH 2023). LSD was reported in Turkey in 2013 from where the disease spread to Balkan region during 2015 and 2016. The first case of LSD in Serbia was recorded in June 2016 in southern part of the country (Kumar 2011; Tageldin et al. 2014; EFSA 2015, 2017; Mercier et al. 2018; Manić et al. 2019). The lumpy skin disease virus (LSDV) was isolated from skin nodules, named SERBIA/Bujanovac/2016 and later successfully used for detection of antibodies against LSDV in modified virus neutralization test (VNT) (Toplak et al. 2017; Samojlović et al. 2019).

Vaccination represents the key factor for LSD control and the most efficient way to stop spreading of the disease (EFSA 2018; EFSA 2020). Currently, live attenuated vaccines (Neethling strain) are available for widespread use. The duration of postvaccinal immunity is still questionable, but it is known that not all vaccinated animals can elicit detectable levels of antibodies and that the level of protection cannot be directly related to serum levels of LSDV antibodies (Tuppurainen et al. 2021). Conducting vaccination on regular basis, yearly, and getting the herd immunity to reach over 80% will provide good overall protection (Tuppurainen et al. 2017). According to vaccine manufacturer’s instruction, calves born to vaccinated cows should be vaccinated at six months of age, but giving the recent research results, that protocol would not provide adequate protection (Agianniotaki et al. 2018). After the LSD outbreak in Serbia in 2016 calves born to immunized cows were vaccinated at six months of age. The LSD was successfully eradicated from Balkan region due to strict control measures and effective vaccination campaign (Manić et al. 2019; EFSA 2020).

Immunity to capripoxviruses is mainly cell-dominated but humoral immune response plays a significant role in defense mechanisms of organism from these viruses as well (Woods 1988; Seet et al. 2003). Subclinically infected animals or animals vaccinated against LSDV often develop low levels of neutralizing antibodies that cannot be detected by available serological tests, however that does not indicate that they are not protected. Antibodies usually elicit around 15 days after vaccination or infection, reaching its peak around day 30 and afterwards the levels of antibodies tend to decrease. (Tuppurainen et al. 2017; Babiuk 2018a). Passive immunity in calves born to cows vaccinated against LSDV persists up to six months (Weiss 1968). Until recently, the persistence of maternally derived antibodies against LSDV in calves born to immunized cows has not been studied. Agianniotaki et al. (2018), monitored the presence of neutralizing antibodies in calves born to immunized cows against LSDV by VNT for 150 days. Similar study was conducted in Serbia where the levels of postvaccinal antibodies in cows and maternally derived antibodies in their calves were compared by different serological methods (Milovanović et al. 2019).

Serological diagnostics of LSDV poses a challenge because following vaccination against LSDV often low or undetectable levels of neutralizing antibodies are present (Babiuk 2018a, b). The gold standard and only recommended serological test by WOAH is VNT (WOAH 2023). The performance of ELISA is similar to VNT, although VNT is slightly more specific (Babiuk et al. 2009; Bowden et al. 2009; Berguido et al. 2022). Comparative examination of post vaccinal antibodies in immunized cattle was performed using commercially available ELISA and modified VNT with satisfactory results (Samojlović et al. 2019).

The objective of this study was to assess passive immunity transfer and duration of maternally derived antibodies against LSDV in calves born to vaccinated cows, as well as to compare the performance of two different serological methods ELISA and VNT for the detection of humoral immune response against LSDV in newborn calves and their vaccinated mothers.

## Materials and methods

### Cattle population, vaccination and sampling

**Fig. 1:**
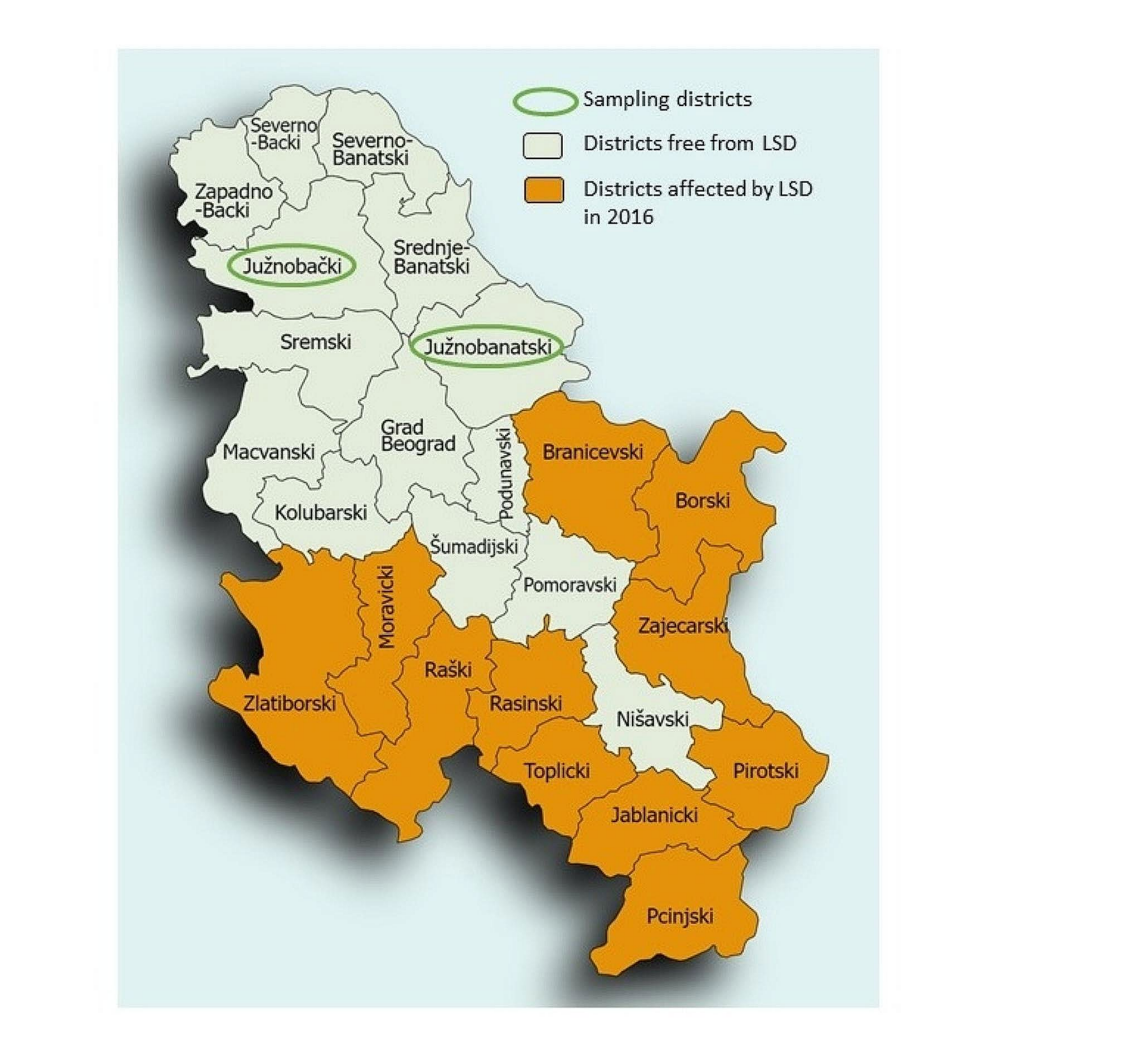
Geographical position of the districts from which the samples were collected, Republic of Serbia (created with http://www.paintmaps.com, Accessed 6th May 2023)

The study was conducted at two dairy farms in Vojvodina province, Serbia, where no cases of LSD were reported during LSD outbreak in Serbia in 2016, in South Backa District (Farm A) and in South Banat District (Farm B) (Fig. 1). All cows in both farms were vaccinated against LSDV in August 2016 and revaccinated in August 2017 by official veterinarians as a part of Programme of measures of health protection of animals in Republic of Serbia. Vaccination of cattle against LSDV in Serbia in 2016 after the LSD outbreak was carried out with vaccine “OBP Lumpy Skin Disease” (Onderstepoort, Biological Products, Onderstepoort, South Africa), while in 2017 vaccination against LSDV was carried out with vaccine “Bovivax LSD-N” (MCI Sante Animale, Mohammedia, Morocco). Both vaccines contained live attenuated LSDV Neethling strain.

Fifteen cows from Farm A expected to calve in September and October 2017 and fifteen cows from Farm B expected to calve in January 2018 were selected to participate in this study. The selection of cows was made according to the approximate calving day with all animals being in good condition and general health. No first calf heifers were included in the study. Thirty blood sera samples from cows were collected on the day of calving. Additionally, colostrum samples were collected on the day of calving from 15 cows on Farm B. Blood sera samples from thirty newborn calves born to the abovementioned cows were collected on days 10, 20, 30, 45, 60, 75, 90, 105 and 120 after birth. A total of 270 blood sera samples from calves were collected. All calves were separated from their mothers and individually kept afterwards. Within two hours after birth calves were bottle-fed 2.5 L of colostrum and afterwards in the first 48 h after calving all calves were fed 2.5 L of colostrum from their mothers two times per day.

### ELISA

For detection of antibodies against LSDV in blood sera and colostrum sera samples, a commercial ELISA kit “ID Screen® Capripox Double Antigen Multi-species”, manufactured by IDvet (France) was used according to manufacturer’s instructions. The same protocol of testing was used for both blood and colostrum sera samples.

### Modified VNT

For detection of neutralizing antibodies against LSDV a modified VNT was used as previously described by Samojlović et al. (2019). The modification of VNT in comparison to the VNT described in OIE Terrestrial Manual (Chap. 3.4.12.) referred to virus strain, cell line used and duration of the test procedure, as well as using of two-fold serial titration of serum against constant dilution of virus (serum titer) instead of using serial titration of virus against constant dilution of serum (neutralizing index) (WOAH 2023).

### Statistical data analyses

Statistical analyses for evaluation of methods ELISA and modified VNT were performed by kappa test (http://epitools.ausvet.com.au/content.php?page=Compare2Tests, Accessed 15th March 2023). An additional comparison of serological methods was performed by McNemar’s x^2^-test (Prism GraphPad). Chi-square and Mann-Whitney U tests (Prism GraphPad) were used for calculating statistical significance between number of positive LSDV antibody samples between farms by serological methods and between antibody levels in colostrum and blood sera samples of vaccinated cows, respectively. A p-value < 0.05 was considered statistically significant.

## Results

Out of 30 tested cow sera samples on the day of calving specific antibodies against LSDV were determined in 63.33% and 73.33% by ELISA and VNT respectively. In calves a total of 270 samples were tested and 39,63% positive samples were detected by ELISA and 35.56% by VNT.

More total positive samples to LSDV antibodies by ELISA were detected on Farm B (47.33%) compared to Farm A where 36.67% positive samples were detected. The same number of positive samples of neutralizing LSDV antibodies was detected by VNT on both farms and was 39,33%. On Farm A it was possible to detect maternal LSDV antibodies in calves for a longer period, even 120 days after birth, while on Farm B the last antibody positive samples were detected 90 days after birth.

No statistically significant difference was found in the number of positive cow sera samples between farms neither by ELISA nor VNT. Ten and twenty days after birth statistically significant difference (*p* < 0.05) was observed in number of positive samples in calves on Farm A comparing to Farm B by ELISA. Also, thirty days after birth statistically significant difference (*p* < 0.01) was observed in number of positive samples in calves on Farm A comparing to Farm B by ELISA. In following sampling time points (45, 60, 75, 90, 105 and 120 days after birth) no statistically significant difference (*p* > 0.05) was found in number of positive samples in calves between farms by ELISA. By VNT no statistically significant difference (*p* > 0.05) was found in number of positive samples in calves between farms in any sampling time points.

### Evaluation of passive immunity transfer against LSDV by ELISA

**Fig. 2:**
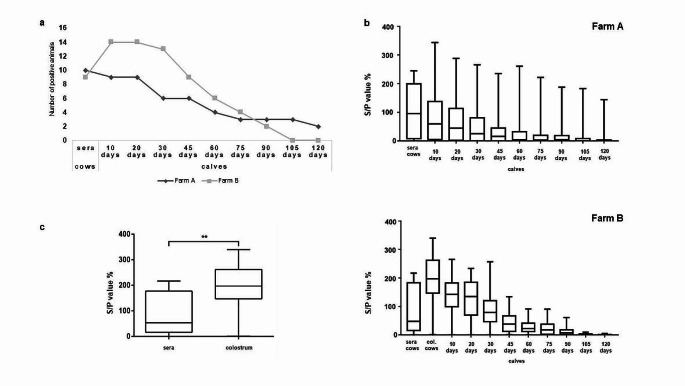
**a **Detection of antibodies against LSDV in cows and calves by ELISA test in both Farms A + B; **b** Average longitudinal LSDV antibody levels in cows and calves by ELISA test in both Farms A + B (S/P% values); **c** Blood sera and colostrum LSDV antibody levels by ELISA from cows in Farm B (**p < 0.01)

By ELISA 19 (63.33%) out of 30 cows tested positive for the presence of LSDV antibodies, while the transfer of antibodies by colostrum was confirmed in 24 (80%) calves 10 days after birth. On Farm B, 13 (86.87%) colostrum samples out of 15 were positive to LSDV antibodies and transfer of antibodies was detected in 14 (93.33%) calves. As shown in Fig. 2a more positive samples in calves were detected on Farm B from 10 to 75 days after birth, while in two last sampling time points on Farm B there were no positive samples detected.

A higher average S/P value in all longitudinal samples was detected on Farm B as well as a higher number of positive samples. The highest S/P values were detected in colostrum samples on Farm B, while sera samples from same cows had much lower average S/P values with statistically significant difference (*p* < 0.01) between LSDV antibody level in colostrum comparing to cow sera (Fig. 2b and c).

### Evaluation of passive immunity transfer against LSDV by modified VNT

**Fig. 3:**
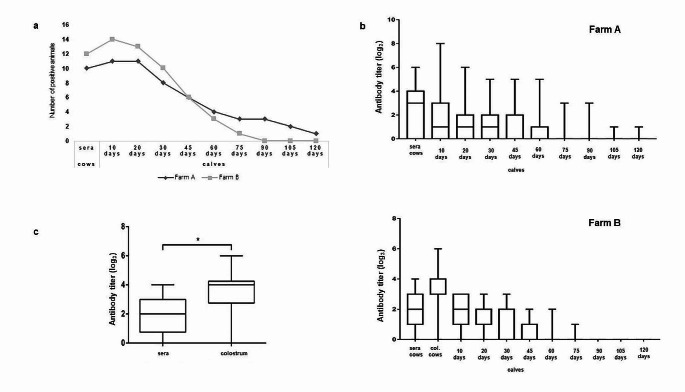
**a **Detection of antibodies against LSDV in cows and calves by VNT in both Farms A + B; **b **Average longitudinal LSDV antibody levels in cows and calves by VNT in both Farms A + B (antibody titer values log2); **c **Blood sera and colostrum LSDV antibody levels by VNT from cows in Farm B (*p < 0.05)

Number of positive sera samples in cows to LSDV antibody detected by VNT was 22 (73.33%) out of 30 tested. Succesful transfer of maternal antibodies was observed in 25 (83.33%) calves 10 days after birth. On Farm B, the same results were obtained by VNT as by ELISA, where 13 (86.87%) colostrum samples out of 15 were positive to LSDV antibodies and transfer of antibodies was detected in 14 (93.33%) calves. The highest number of calves with positive neutralizing antibody finding was detected 10 days after birth (83.33%). Afterwards, the number of positive calves stared to decrease. (Fig. 3a).

Higher average titer values were observed on Farm A (Fig. 3b). On Farm B statistically significant diffrence (*p* < 0.05) in LSDV antibody titer in colostrum comparing to cow sera was observed (Fig. 3c) with higher average antibody titer values detected in colostrum.

### Comparative evaluation of methods ELISA and modified VNT

The results of comparartive testing did not match in 40 out of 300 tested samples. Out of 40 samples which results did not match, 14 samples were from calves on Farm A, while 26 samples were from Farm B, 3 samples from cows and 23 samples from calves. Positive LSDV antibody samples were detected in 126 and 118 samples by ELISA and VNT respectively, while 174 negative samples were detected by ELISA and 182 by VNT. The calculated kappa coefficient was 0.724 indicating substantial agreement between two tests. Moreover, McNemar’s x^2^-test showed no statistically significant difference between the two tests (p value 0.2684).

## Discussion

In this paper we present the results of longitudinal study of the presence and duration of passive immunity in calves born to cows vaccinated against LSDV and the results of comparative examination of maternally derived LSDV antibodies by two different serological methods, commercially available ELISA and modified VNT. On Farm A, a higher number of positive samples was detected by VNT, whilst on Farm B a higher number of positive samples was detected by ELISA method. The variations in number of positive samples on both farms by different serological methods could be the consequence of time difference between the date of vaccination and the date of calving on farms, as well as the fact that different kind of antibodies are detected by different serological tests. Similar results can be seen in the study of colostral LSDV antibody transfer, where the higher number of positive samples was also detected by VNT in cow and calf sera (Milovanović et al. 2019).

Field study described by Hunter and Wallace (2001), showed that 3 of 30 tested cows did not seroconvert after LSDV vaccination but were protected after a challenge. One of those three cows did not provide passive immunity to its calf that died of LSDV infection at the age of 2 weeks. In our study two cow sera samples from each farm tested negative for LSDV antibodies on the calving day, but the calves still had detectable levels of antibodies. In three calves and three cows the positive results were obtained only by VNT. In one calf, the positive results were obtained only by ELISA and despite very high S/P values the VNT was negative. Milovanović et al. (2019), could not detect LSDV antibodies in all samples by all three different serological methods (VNT, ELISA, IFAT). The highest number of calves with positive antibody finding by both methods could be detected 10, 20 and 30 days after birth, afterwards the numbers began to decline. On Farm B, 90 days after birth there were no positive antibody results in calves, whilst on Farm A at the last sampling period of 120 days after birth, only two calves had positive antibody results by ELISA. These results correlate with the results of other authors where 90 days after birth one third of the calves tested positive and 120 days after birth no calves tested positive (Agiannotaki et al. 2018).

It was reported that the presence of maternal antibodies can affect the full potential of active immunity in calves up to 6 months of age. Literature data suggests that calves of vaccinated or naturally infected cows, and those vaccinated before the age of six months can have inadequate protection precisely because of the interference of maternal immunity with the vaccine strain of the virus (Tuppurainen and Oura 2012; Babiuk 2018a). Looking at our results, we can report that the majority of the tested calves had undetectable levels of LSDV antibodies 3 months after birth, much earlier than suggested in the literature. Considering the fact that the role of protective immunity against LSD was confirmed in several studies in cattle where no detectable levels of antibodies were reported (Kitching 1986; Bowden et al. 2009) and the lack of data on passive immunity in calves (Tuppurainen et al. 2017), the results of this study can help to better understand the dynamics of passive immunity transfer and duration of maternally derived antibodies.

The higher number of positive results of LSDV antibodies in cow colostrum compared to blood sera confirms the fact that antibodies are present in colostrum at high concentration that is not always detectable in the sera. On Farm B the presence of antibodies in colostrum was determined in 13 samples by both methods, while in sera the presence of antibodies was determined in 9 and 12 samples by ELISA and VNT, respectively. Also, the antibody titers were much higher in colostrum than in sera. The same results were seen in the study by Agianniotaki et al. (2018). The process of colostrogenesis starts several weeks before partus to create IgG rich colostrum which can lead to the drop of IgG concentration in the sera (Baumrucker et al. 2016).

To compare the performance of two serological methods for the detection of antibodies against LSDV, we used longitudinal sera samples from vaccinated cows and their calves. In a comparative examination of blood serum samples of vaccinated cows and their calves by ELISA and VNT methods for the detection of antibodies against LSDV the value of the kappa coefficient was 0.7239, which shows substantial, good agreement of the compared methods. The number of samples that did not match in a comparative examination of ELISA and VNT in longitudinal serum samples from vaccinated cows and their calves was four times higher compared to our previous study with longitudinal serum samples of vaccinated cows (Samojlović et al. 2019). Similar results from ELISA/VNT performance comparison were obtained in the study by Krešić et al. (2020), with kappa coefficient of 0.834 (very good, excellent agreement). McNemar’s x^2^-test indicated no statistically significant difference in performance of ELISA and modified VNT which is in accordance with the results reported by Fay et al. (2022).

## Conclusions

The LSDV maternal antibody levels were below detectable level in the majority of the tested calves 3 months after vaccination, so it would be very useful to reconsider the optimal age for vaccination of calves from vaccinated cows to achieve the highest possible level of protection against LSDV. Moreover, significantly higher levels of antibodies were found in the colostrum compared to blood sera of vaccinated cows indicating the good potential for LSDV maternal antibody transfer if the calves are fed with colostrum immediately after birth. Both tests, commercially available ELISA and modified VNT showed good performance in detection of LSDV antibodies, but further modifications are required for these assays to reach peak performance and to become valuable and irreplaceable tool in the fight against LSDV through screening, surveillance and vaccination monitoring.

## Data Availability

No datasets were generated or analysed during the current study.
